# Rational Design of Double Hole Transfer Layers for Efficient CdTe Nanocrystal Solar Cells

**DOI:** 10.3390/nano16040239

**Published:** 2026-02-12

**Authors:** Zheng Zhou, Xinyi Wang, Jielin Huang, Qichuan Huang, Donghuan Qin

**Affiliations:** 1 School of Materials Science and Engineering, South China University of Technology, Guangzhou 510640, China; 202320118465@mail.scut.edu.cn (Z.Z.); 202420119282@mail.scut.edu.cn (X.W.); 202230271209@mail.scut.edu.cn (J.H.); hqcscut@outlook.com (Q.H.); 2 State Key Laboratory of Luminescent Materials & Devices, Institute of Polymer Optoelectronic Materials & Devices, South China University of Technology, Guangzhou 510640, China

**Keywords:** CdTe, solar cells, nanocrystalline

## Abstract

Energy losses induced by inefficient charge transfer and large energy-level offsets at the interface limit the efficiency of CdTe nanocrystal (NC) solar cells. In this work, organic poly(triaryl amine) (PTAA) and inorganic CuI which form double hole transport layers (HTLs) are first proposed to improve the charge transfer capability of hole transport layers (HTLs) and reduce the band offset at the interface of CdTe NCs. The introduced CuI improves carrier mobility, while PTAA reduces interface recombination and reinforces the inner built-in field, resulting in low energy loss from the CdTe NC active layer to the contact electrode. Photovoltaic devices using these modified back contacts show increases in both open-circuit voltage and short-circuit current, compared to a controlled device without HTL. The CdTe NCs utilizing CuI-PTAA double HTLs demonstrate a high power conversion efficiency (PCE) of 7.36%. High stability is also demonstrated, with PCE loss being less than 5% after tracking for 30 days. This work provides an effective way to minimize energy loss at the interface of the back contact in inverted CdTe NCs solar cells, by incorporating proper hole transfer layer design.

## 1. Introduction

Recent advances in CdTe nanocrystal (NC) solar cells have garnered substantial research interest because of their cost-effective fabrication protocols; these include minimal material consumption, and compatibility with large-scale roll-to-roll printing manufacturing [1,2,3,4,5,6]. For inverted-structure CdTe NC solar cells, the sandwiched architecture between the cathode and anode typically comprises an n-type acceptor layer and a photoactive layer, wherein light absorption and charge generation predominantly occur. Optimizing carrier transport dynamics at the interface between the CdTe NC layer and the back-contact electrode is widely recognized as a pivotal strategy for boosting device photovoltaic performance [2,7]. Given that pristine CdTe materials inherently exhibit high resistivity and low hole concentration, it is imperative to implement tailored modification approaches to elevate surface hole density and mitigate interfacial contact barriers [8,9]. In previous studies, two primary strategies have been proposed to tackle this critical issue: one entails the introduction of copper ion doping to enhance intrinsic hole concentration, the other involves the incorporation of a dedicated hole-transporting layer to decrease interfacial charge extraction barriers [10,11,12,13,14].

The copper ion doping strategy modulates the intrinsic electronic properties of CdTe NCs by engineering shallow acceptor levels within the bandgap, thereby facilitating efficient hole excitation and lateral migration throughout the photoactive layer. In contrast, the integration of a hole-transporting layer enables construction of a favorable energy-level alignment at the CdTe/electrode interface, which serves to minimize parasitic energy loss during the charge extraction process [15,16,17]. It should be noted that the oxygen atoms present in MoO_x_ and other HTLs may form n-type CdO at the cadmium telluride interface, which could degrade device performance. Therefore, exploring the synergistic integration of these two complementary strategies has emerged as a highly promising research direction, offering the potential to circumvent their respective limitations and fully exploit the performance capabilities of inverted CdTe NC solar cells.

Prior work by Yang et al. [18] explored organic hole-transporting materials (HTMs) for the surface passivation of CdTe NCs, employing the device architecture FTO/TiO_2_/CdTe/Spiro-OMeTAD(2,2′,7,7′-tetrakis[*N*,*N*-di(4-methoxyphenyl)amino]-9,9′-spirobifluorene)/Au. Their findings revealed that Spiro-OMeTAD modification induces interfacial dipole effects, which in turn mitigate back-contact recombination losses. Subsequently, our research group reported a novel, highly cross-linkable conjugated polymer poly(diphenylsilane-co-4-vinyl-triphenylamine) (Si-TPA) as a HTM for inverted CdTe NC solar cells with the configuration ITO/ZnO/CdSe/CdTe/Si-TPA/Au. This material integration enabled simultaneous enhancements in open-circuit voltage (*V*_OC_), short-circuit current density (*J*_SC_), and fill factor (FF), culminating in a high power conversion efficiency (PCE) of 8.34%. Notably, experimental studies have established that CdTe NCs exhibit a work function in the range of 5.2–5.3 eV, concomitant with an intrinsic cadmium-rich surface [19]. The deliberate selection of organic HTMs with a highest occupied molecular orbital (HOMO) energy level of ~5.3 eV has been shown to passivate surface defects, suppress carrier recombination, and augment charge collection efficiency, as corroborated by our follow-up investigations [20]. Nevertheless, the intrinsically low electrical conductivity of most organic HTMs, in conjunction with the high resistivity of pristine CdTe, imposes a bottleneck that precludes further performance optimization when only an organic hole transport layer (HTL) is incorporated into the device architecture. For CdTe thin-film solar cells fabricated via close-space sublimation, interfacial engineering strategies involving the deposition of a thin copper layer at the back contact have been demonstrated to effectively boost localized hole concentration and facilitate the formation of ohmic contact [21,22]. Extensive research has further verified that copper layer deposition on CdTe surfaces yields an increase in hole density and a reduction in back-contact barrier height, thereby enabling superior device performance metrics [23]. However, solution-processable CdTe NCs are characterized by abundant grain boundaries within their microstructure; direct copper deposition onto such NC films triggers rapid copper atom diffusion along these grain boundaries. In severe cases, this phenomenon can even induce p-n junction inversion, leading to a marked deterioration in device stability [24,25]. Conversely, the incorporation of copper salt doping has proven to be an effective strategy for inhibiting copper diffusion kinetics while maintaining stable device performance [26]. This approach has been substantiated by recent research endeavors [27]. Therefore, the exploration of synergistic coupling between organic HTMs and copper salt doping, to harness the respective merits of both modification strategies, represents a compelling and high-impact research direction for advancing CdTe NC solar cell technology.

To address the limitation of a single hole transport layer, a novel PTAA/CuI material system is developed as a double hole transport layer. The p-type PTAA, with a highest occupied molecular orbital (HOMO) level of approximately 5.2 eV, exhibits excellent compatibility with the CdTe NCs layer [28,29,30]. This compatibility can notably enhance the extraction of hole carriers from the CdTe NC layer. Moreover, CuI prepared by thermal evaporation is a p-type semiconductor material with excellent electrical properties. This material has been explored as a back contact, helping to ensure favorable band alignment, while the copper ions contained within it can diffuse into the cadmium telluride layer. This is beneficial, as the diffusion of Cu into the CdTe NCs reduces the contact resistance and increases the hole concentration [31]. Through the optimization of device fabrication parameters, such as the thickness of the PTAA film and the annealing temperature of the hole transport layer, a high power conversion efficiency of 7.36% is achieved. This value is significantly higher than that of the control device lacking any hole layer, which has a short-circuit current density of 19.12 mA/cm^2^, an open-circuit voltage of 0.50 V, and a PCE of 5.05%. Based on the space-charge-limited current (SCLC) measurement, the optimized device demonstrates a hole mobility of 1.87 × 10^−3^ cm^2^/(V·s), which surpasses that of the control device (2.52 × 10^−4^ cm^2^/(V·s)). The capacitance–voltage (*C*-*V*) measurement reveals the presence of a higher built-in internal field, attributed to the formation of a dipole layer between the PTAA and CdTe NC layer. This phenomenon facilitates hole collection and increases the *V*_OC_, which is consistent with the results of the current density–voltage (*J*-*V*) measurement. Consequently, it is demonstrated that the incorporation of CuI/PTAA as the HTL can substantially enhance the performance of solution-processed CdTe NC solar cells. This research presents a novel approach for the development of high-performance CdTe NC–based solar cells.

## 2. Experiment Procedure

p-type CdTe nanocrystals, n-type CdSe nanocrystals, and ZnO precursors were prepared in accordance with well-established synthesis protocols reported in the literature [17,32,33,34]. The detailed synthesis procedures for cadmium telluride and cadmium selenide nanocrystals are provided in the Supplementary Materials. Poly [bis(4-phenyl)(2,4,6-trimethylphenyl)amine] (PTAA) was purchased from Derthon Optoelectronic Material Science & Technology Co., Ltd. (Shenzhen, China) and utilized as received without further purification. Copper(I) iodide (CuI) was acquired from Shanghai Aladdin Biochemical Technology Co., Ltd. (Shanghai, China). The photovoltaic device films were fabricated via sequential spin-coating and sintering under ambient atmospheric conditions, using procedures based on previously documented methodologies, modified slightly.

Specifically, a zinc-containing precursor was first deposited onto indium tin oxide (ITO)-coated substrates, followed by annealing at 400 °C for 10 min to remove residual solvents and enable formation of a dense ZnO thin film. Subsequently, two consecutive layers of CdSe NC solution were spin-coated onto the ZnO film to achieve a total thickness of ~70 nm. The as-deposited CdSe film was then annealed at 350 °C for 30 min to construct the n-type active layer. Next, five layers of p-type CdTe NCs were deposited by spin-coating onto the CdSe NC layer at 1100 rpm for 20 s. Post-deposition, the CdTe film was treated with a saturated cadmium chloride (CdCl_2_) methanol solution, followed by annealing at 350 °C for 30 min to enhance crystallinity and optimize interfacial properties. It should be noted that the formation of a compositionally graded CdSe_x_Te_1−x_ interfacial layer during device fabrication has been established as a key contributor to enhanced device performance, a finding consistent with prior observations from our group in which a similarly graded CdSe/CdTe heterointerface was identified [20]. Nevertheless, the potential adverse effects associated with selenium residues resulting from incomplete interdiffusion require careful mitigation [35].

For the fabrication of the hole transport layer, a PTAA solution in chlorobenzene (concentration: 0–10 mg/mL) was spin-coated onto the CdTe layer at 2000 rpm, resulting in a film thickness of 0~20 nm. The samples were subsequently annealed at 120 °C for 10 min to facilitate PTAA cross-linking and improve film compactness. Thereafter, a 5 nm thick CuI layer was thermally evaporated through a shadow mask, defining an active device area of 0.16 cm^2^. Finally, an ~80 nm thick gold (Au) layer was deposited via thermal evaporation as the top electrode, to complete the device structure.

## 3. Results and Discussion

**Figure 1:**
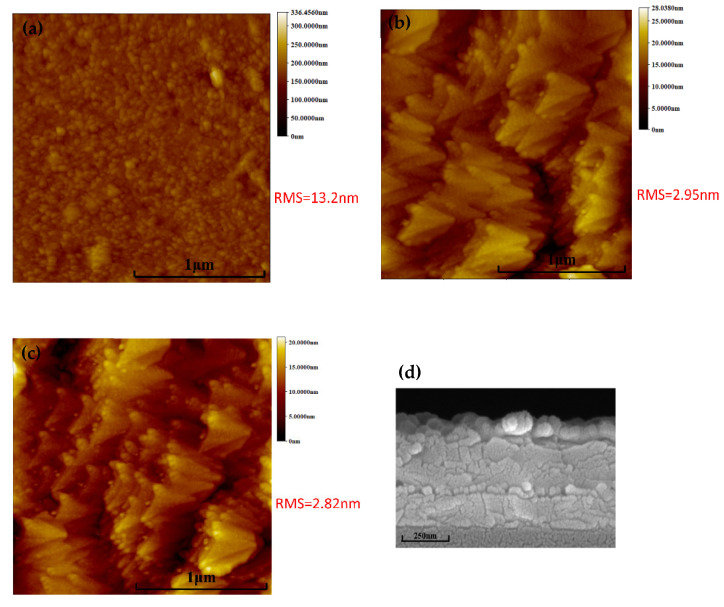
AFM images of ITO/ZnO/CdSe/CdTe thin films: (**a**) without any HTLs, (**b**) with CuI HTL, (**c**) with PTAA/CuI HTLs. (**d**) Cross-section SEM image of ITO/ZnO/CdSe/CdTe.

To investigate the influence of diverse HTLs on the morphological properties of CdTe nanocrystal (NC) thin films, CuI and PTAA were deposited onto the ITO/ZnO/CdSe/CdTe substrate via thermal evaporation or spin-coating, respectively. The morphological evolution of CdTe samples modified by different HTLs is systematically illustrated in Figure 1. As revealed by atomic force microscopy (AFM) characterization (Figure 1a), the pristine (untreated) CdTe NC surface exhibits a smooth topography, with only a sparse distribution of discrete particles localized exclusively at the grain boundaries. This observation is consistent with the typical morphological features of solution-processed CdTe NC films reported in previous studies [17], where minimal grain boundary defects are preferred for decreasing interface defects. Upon the introduction of CuI or PTAA HTLs, the pinholes inherently present in the CdTe NC film are effectively filled by the HTL materials. This filling effect is particularly critical for mitigating interface defects—a key factor that often induces carrier recombination at the active layer/HTL interface. Furthermore, quantitative AFM analysis provides insights into the surface roughness of the modified films. The root mean square (RMS) roughness values of the films are determined to be 13.2 nm for the pristine (control) CdTe sample, 2.95 nm for the CdTe/CuI composite, and 2.82 nm for the CdTe/PTAA composite. This trend indicates that the integration of HTLs—especially PTAA—significantly enhances the surface smoothness of the ITO/ZnO/CdSe/CdTe/HTL stack. A smoother active layer surface is thermodynamically favorable for reducing interfacial trap states and minimizing carrier recombination losses, thereby facilitating more efficient carrier collection at the electrode. Cross-sectional scanning electron microscopy (SEM) images (Figure 1d) further confirm that the CdTe NC film maintains a homogeneous and compact microstructure, with a thickness of approximately 400 nm after HTL deposition. This compactness is crucial for preventing shunt current pathways and ensuring effective light absorption within the active layer, which collectively contributes to the overall photovoltaic performance of the device.

**Figure 2:**
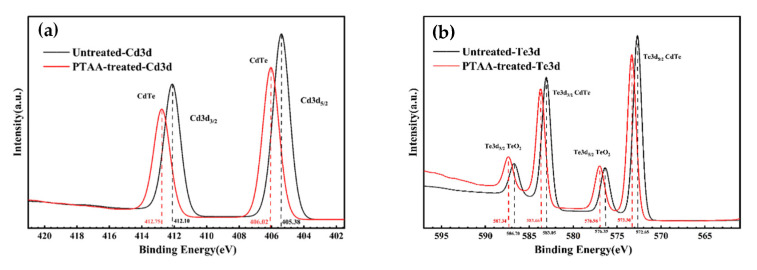
The narrow XPS scan of CdTe NC thin films for (**a**) Cd 3d and (**b**) Te 3d.

To further elucidate the interfacial chemical bonding interactions between CdTe NC thin films and diverse hole transport layers (HTLs), X-ray photoelectron spectroscopy measurements were obtained. Figure 2 presents the high-resolution XPS narrow-scan spectra of CdTe NC thin films in two distinct configurations: without any HTL (pristine), and with PTAA HTL. The full-scan XPS spectra of samples with different HTLs, which confirm the elemental composition of the film systems, are provided in Figure S1. The quantitative results of the observed binding energies for Cd 3d and Te 3d core levels are summarized in Table 1. For the Cd 3d core level, notable differences in binding energy were observed among the samples. Specifically, the Cd 3d_5/2_ and Cd 3d_3/2_ binding energies of the CdTe/PTAA composite film were determined to be 406.02 eV and 412.75 eV, respectively. In contrast, the pristine CdTe film and CdTe/CuI composite film exhibited identical Cd 3d binding energies, with Cd 3d_5/2_ at 405.38 eV and Cd 3d_3/2_ at 412.10 eV. This comparison indicates a distinct 0.6 eV increase in the Cd 3d peaks in the PTAA-modified sample, relative to the other configuration. An analogous trend was observed for the Te 3d core level: the Te 3d binding energies in the CdTe/PTAA film also exhibited an increase relative to the pristine samples, as illustrated in Figure 2b and corroborated by the quantitative data in Table 1; this implies formation of Cd-N chemical bonding at the CdTe/PTAA interface, which modifies the vacuum level, leading to an upward shift in the electron binding energy of interfacial Te and Cd atoms [36]. The establishment of Cd-N covalent bonds enables tight interfacial adhesion between PTAA and the CdTe surface—an interaction that plays a pivotal role in passivating interfacial trap states and suppressing the recombination of free electron–hole pairs. With respect to the Cu element, the presence of Cu 2p peaks in the XPS spectra of the CdTe/CuI composite film (as shown in Figure S1) is attributed exclusively to the CuI HTL, confirming the successful deposition of CuI on the CdTe NC surface. Additionally, quantitative XPS analysis revealed that the atomic ratio of Te to Cd (Te/Cd) was approximately 0.58 across all CdTe NC film samples (pristine, CdTe/CuI, and CdTe/PTAA). This Te-deficient (Cd-rich) composition can be rationalized by the post-deposition CdCl_2_ treatment applied to the CdTe NC films: even after rinsing with methanol, residual CdCl_2_ remains adsorbed on the CdTe NC surface, leading to an excess of Cd in the overall film composition. This observation is consistent with previous reports on CdCl_2_-treated CdTe thin films in which surface Cd enrichment has been shown to be a common phenomenon arising from incomplete removal of the CdCl_2_ passivation agent [37].

To gain deeper insights into the charge transport properties of CdTe NCs with different HTLs, hole-only devices with the architecture ITO/CdTe (200 nm)/HTL/Au (80 nm) were fabricated and systematically investigated. The space-charge-limited current (SCLC) method was employed to calculate the hole carrier mobility of the CdTe NCs thin films, based on the following equation [38]:

$$J=\frac{9}{8}\varepsilon_{0}\varepsilon_{r}\mu_{p}\frac{{\left(V-V_{bi}\right)}^{2}}{L^{3}}$$

**Figure 3:**
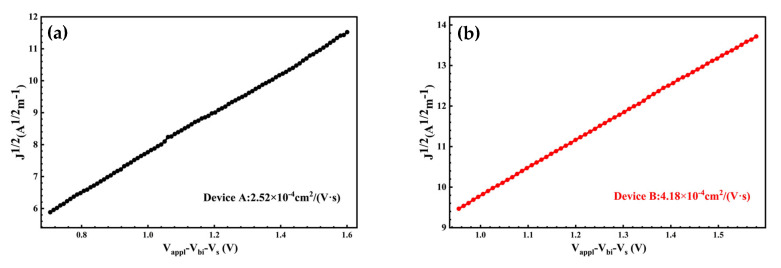

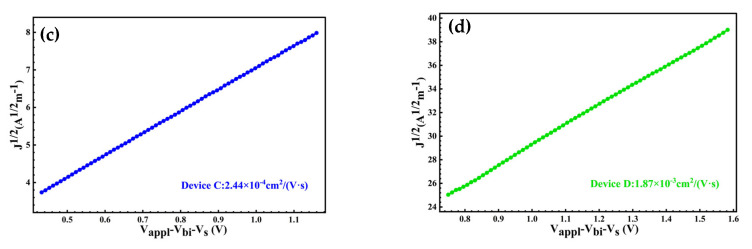
SCLC measurements of hole-only devices. (**a**) ITO/CdTe/Au; (**b**) ITO/CdTe/CuI/Au; (**c**) ITO/CdTe/PTAA/Au; and (**d**) ITO/CdTe/PTAA/CuI/Au.

Herein, *ε*_0_ denotes the permittivity of free space, *ε_r_* = 9.8 is the relative dielectric constant of CdTe, *L* represents the thickness of the CdTe NCs film, *μ_p_* is the hole mobility, *V* is the applied voltage, and *V_bi_* is the built-in voltage. As shown in Figure 3, the mobility of controlled devices is 2.52 × 10^−4^ cm^2^/(V·s). Contrarily, higher mobility values are obtained in the case of the CdTe device with HTL. The mobilities of CdTe NCs thin film with HTL show improved values of 4.18 × 10^−4^ cm^2^/(V·s), 2.44 × 10^−4^ cm^2^/(V·s) and 1.87 × 10^−3^ cm^2^/(V·s) for ITO/CdTe/CuI/Au, ITO/CdTe/PTAA/Au and ITO/CdTe/PTAA/CuI/Au respectively. Therefore, devices with HTL show higher hole mobility, decreasing the energy barrier for the hole transfer from CdTe to Au; thus, carrier collection efficiency is improved in this case.

**Figure 4:**
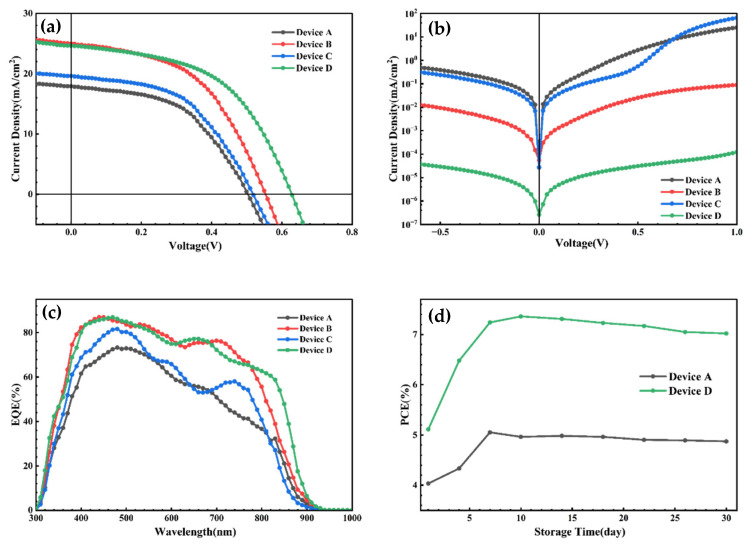
*J*-*V* curves for four kinds of CdTe NC solar cells. (**a**) Under 100 mW cm^−2^ (AM 1.5 G) illumination. (**b**) Under dark and (**c**) corresponding EQE. (**d**) Stability in ambient condition.

The incorporation of a HTL into solution-processed CdTe NC solar cells induces reorganization of the CdTe NC film at the interface, thereby enhancing carrier collection efficiency at the back contact. Solar cells with the device architecture ITO/ZnO/CdSe/CdTe/HTL/Au were fabricated and systematically characterized. Initially, single HTL-based devices were investigated to determine the optimal annealing temperature (Tables S1 and S2). For the ITO/ZnO/CdSe/CdTe/CuI/Au device, the optimal annealing temperature was found to be 80 °C; both lower and higher annealing temperatures resulted in deteriorated device performance. In contrast, the optimal annealing temperature for the ITO/ZnO/CdSe/CdTe/PTAA/Au device was determined to be 120 °C. Figure 4a presents the current density–voltage (*J*-*V*) curves for the fabricated devices, with detailed photovoltaic parameters summarized in Table 2. The control device (Device A) without HTL exhibited a short-circuit current density (*J*_SC_) of 19.12 mA/cm^2^, an open-circuit voltage (*V*_OC_) of 0.50 V, and a fill factor (FF) of 52.82%, corresponding to a power conversion efficiency (PCE) of 5.05%. In comparison, the CuI-based HTL device (Device B) demonstrated significantly enhanced performance, with a *J*_SC_ of 24.99 mA/cm^2^, a *V*_OC_ of 0.55 V, an FF of 49.76%, and a resultant PCE of 6.84%. When PTAA was employed as the HTL (Device C), the device achieved a *J*_SC_ of 19.61 mA/cm^2^, a *V*_OC_ of 0.52 V, an FF of 50.11%, and a PCE of 5.11%. We propose that the PTAA/CuI double-HTL configuration outperforms either of the single-HTL configurations, as it integrates the advantageous properties of both individual HTLs. The champion device based on the PTAA/CuI double HTL delivered a *J*_SC_ of 24.62 mA/cm^2^, a *V*_OC_ of 0.62 V, an FF of 48.23%, and a PCE of 7.36%. Consequently, the PCE of the PTAA/CuI-based device was enhanced by 45%, compared to the control device. Device D exhibited a dark current density that was one order of magnitude lower than that of the others (Figure 4b), indicating that the introduction of HTLs effectively suppresses the leakage current at the anode. The external quantum efficiency (EQE) spectra depicted in Figure 4c provide insights into the mechanism underlying the improved *J*_SC_ of HTL-integrated devices. Relative to the control device (Device A), Devices B, C, and D all showed a significant enhancement in EQE across almost the whole wavelength range from 400 to 900 nm. This improvement is attributed to the enhanced carrier collection efficiency facilitated by the HTL, which passivates the interfacial defects of the CdTe NC film and thereby promotes efficient carrier extraction. It is well established that the device lifetime constitutes a critical challenge in solution-processed CdTe NC solar cells, which are partially affected by trap states originating from the back contact. Notably, both the PTAA/CuI-integrated devices and the control device exhibited excellent long-term stability (Figure 4d). The device performance was observed to improve after 3 days of storage, and it remained nearly unchanged over the subsequent 30-day measurement period. This enhanced stability is presumably associated with the diffusion of Se and Te into the active layer, which reduces bulk defects and facilitates the formation of a homogeneous CdSeTe alloy.

**Figure 5:**
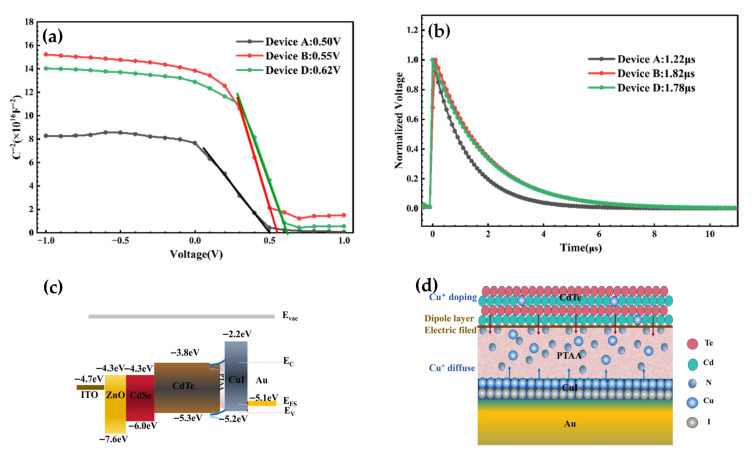
(**a**) Mott–Schottky *C*-*V* curve. (**b**) Transient photovoltage (TPV) and (**c**) band alignment of CdTe NC solar cells. (**d**) A schematic of energy levels influenced by a dipole layer of PTAA and CuI at the CdTe/Au interface.

To gain more insight into the effect of HTL layers on the CdTe NC solar-cell performance, capacitance–voltage (*C*-*V*) analysis is carried out by increasing bias voltage at a constant frequency of 1000 Hz to investigate the built-in electric field of the NC solar cells. As shown in Figure 5a, *C*-*V* curves are plotted based on the Mott–Schottky equation [7]:

$$C^{-2}=\frac{2(V_{bi}-V)}{{A^{2}q\varepsilon }_{0}\varepsilon_{r}N_{A}}$$

where the parameters *A*, *ε*, *ε*_0_, *V_bi_* and *N_A_* are the device active area, relative dielectric constant (10.6), vacuum permittivity, built-in potential and net acceptor concentration, respectively. The *V_bi_* can be extracted at a forward bias from the extrapolated intersection of the slope and x axis. The *V_bi_* of the Device C (PTAA/CuI bi HTL layers) is 0.62 V, which is significantly higher than 0.50 V for the controlled device. The *V_bi_* values agree well with the *V*_OC_ values from the *J*-*V* curves. To further examine the effect of PTAA HTL on the carrier recombination of NC solar cells, the charge recombination lifetime is investigated by transient photovoltage (TPV). The charge recombination processes are studied by tracking the transient open-circuit voltage related to charge population perturbation. As shown in Figure 5b, Device D shows a charge recombination lifetime of 1.78 μs, longer than the 1.22 μs for the controlled device, suggesting that the charge recombination is low for the PTAA/CuI device. As presented in Figure 5c,d, the formation of the dipole layer connecting the CdTe active layer and contact electrode causes a vacuum energy level shift, and thus facilitates hole extraction due to the reduced hole injection barrier. Moreover, the diffusion of Cu^+^ from CuI to CdTe NC active layer increases the concentration of interface holes, decreasing the energy barrier; correspondingly, a high output photocurrent/voltage is expected.

## 4. Conclusions

In summary, we propose a strategy to reduce harmful carrier recombination at the CdTe NC/electrode interface by introducing a PTAA/CuI double HTL between the CdTe NC film and the contact electrode. A dipole layer is formed at the CdTe NC/PTAA interface via the bonding between Cd^2+^ and N^3−^, which significantly improves the open-circuit voltage of the device. Moreover, the presence of CuI atop PTAA induces the diffusion of Cu^+^ into the CdTe NC active layer, which enhances the p^+^-doped interface and reduces the energy barrier between the CdTe NC film and the contact electrode. These synergistic effects lead to remarkable improvements in both *V*_OC_ and short-circuit current density (*J*_sc_), enabling the fabrication of high-efficiency solution-processed CdTe NC solar cells. This work demonstrates that the double HTL strategy can be integrated into future manufacturing systems for low-cost solution-processed CdTe NC solar cells.

## Figures and Tables

**Table 1:** Summarized XPS peaks (eV) and atomic contents of Cd and Te elements for CdTe NC films with or without PTAA.

CdTe Thin Film	Cd 3d_5/2_	Cd 3d_3/2_	Te 3d_5/2_	Te 3d_3/2_	Cd/Te
Untreated	405.38	412.10	572.65	583.05	~1.73:1
With PTAA	406.02	412.75	573.30	583.66	~2.00:1

**Table 2:** Summary of the photovoltaic parameters of the best NC solar cells with different HTLs.

Device	Device Architecture	*V*_OC_ (V)	*J*_SC_ (mA cm^−2^)	FF (%)	PCE (%)	R_s_ (Ω cm^2^)	R_sh_ (Ω cm^2^)
A	ITO/ZnO/CdSe/CdTe/Au	0.50	19.12	52.82	5.05	16.82	228.53
B	ITO/ZnO/CdSe/CdTe/CuI/Au	0.55	24.99	49.76	6.84	9.23	265.37
C	ITO/ZnO/CdSe/CdTe/PTAA/Au	0.52	19.61	50.11	5.11	15.37	285.40
D	ITO/ZnO/CdSe/CdTe/PTAA/CuI/Au	0.62	24.62	48.23	7.36	8.41	317.62

## Data Availability

The original contributions presented in this study are included in the article and the Supplementary Materials. Further inquiries can be directed to the corresponding authors.

## Supplementary Materials

The following supporting information can be downloaded at: https://www.mdpi.com/article/10.3390/nano16040239/s1, Figure S1: This XPS full-scan spectra of CdTe NCs thin film with or with HTL; Table S1: Summarized device performance with PTAA under different temperature treated; Table S2: Summarized device performance with CuI under different temperature treated.
