# Supplementary material for: Rational Design of Double Hole Transfer Layers for Efficient CdTe Nanocrystal Solar Cells
Source: Nanomaterials (Basel). 2026 Feb 12;16(4):239. doi: 10.3390/nano16040239 (PMC12942899; doi:10.3390/nano16040239)
Supplement: Supplementary file 1 [file nanomaterials-16-00239-s001.zip › nanomaterials-4134527-supplementary.pdf]

Rational Design of Double Hole Transfer Layers for Efficient CdTe Nanocrystal Solar Cells

Zheng Zhou [1], Xinyi Wang [1], Jielin Huang [1], Qichuan Huang [1], and Donghuan Qin [1,2*]

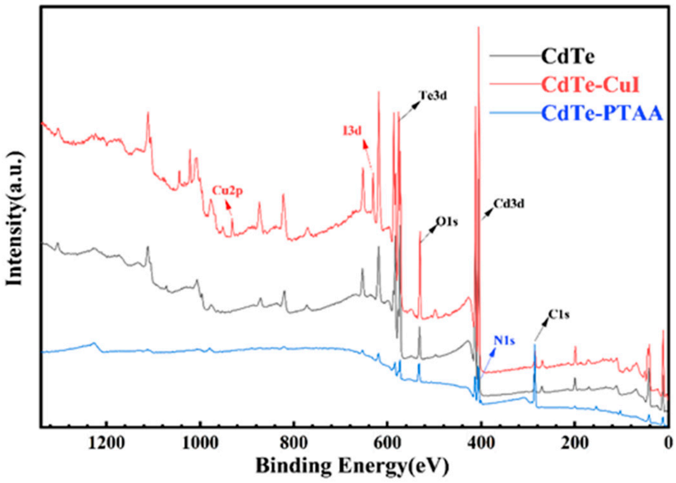

Figure S1 This XPS full-scan spectra of CdTe NCs thin film with or with HTL

[1] School of Materials Science and Engineering, South China University of Technology, Guangzhou 510640, China; 202320118465@mail.scut.edu.cn (Z.Z.); 202420119282@mail.scut.edu.cn (X.W.); 202230271209@mail.scut.edu.cn (J.H.); hqcscut@outlook.com (Q.H.)

[2] State Key Laboratory of Luminescent Materials & Devices, Institute of Polymer Optoelectronic Materials & Devices, South China University of Technology, Guangzhou 510640, China

* Correspondence: qindh@scut.edu.cn (D.Q.); Tel.: +86-020-8711-4346 (D.Q.)

Table S1 Summarized device performance with PTAA under different temperature treated

**Condition**	***V*oc (V)**	***J*sc (mA/cm²)**	**FF (%)**	**PCE (%)**
60	0.51	17.87	47.29	4.31
90	0.51	19.54	49.77	4.96
120	0.52	19.61	50.11	5.11
150	0.50	18.54	45.95	4.26

Table S2 Summarized device performance with CuI under different temperature treated

**Temperature (℃)**	***V*oc (V)**	***J*sc (mA/cm²)**	**FF (%)**	**PCE (%)**
50	0.55	21.67	51.01	6.08
80	0.55	24.99	49.76	6.84
110	0.56	20.03	45.82	5.14
140	0.53	17.78	44.38	4.18

Equipment information

The surface morphology of the thin film was characterized by atomic force microscopy (AFM) using a CSPM 5500 Scanning Probe Microscope in tapping mode. Elemental composition of the surface was analyzed via X-ray photoelectron spectroscopy (XPS) on a Thermo Scientific ESCALAB Xi+ system. Electrical performance of the solar cells was evaluated with a Keysight 2400 Source Meter under AM 1.5 G illumination, provided by an HGSS-50W solar simulator. To further investigate the spectral response, external quantum efficiency (EQE) was measured using an Enlitech QE-R3011 Spectral Response/Quantum Efficiency test system. Capacitance–voltage (*C*–*V*) measurements were performed in the dark at 1000 Hz over a bias range from −1.0 V to 1.0 V using a Keysight E5061B Network Analyzer for device physics analysis. Finally, transient photovoltage (TPV) was recorded with a Zolix OmniFluo measurement system.

Nanocrystals Material Synthesis Method

Under a nitrogen atmosphere, 92 mg of tetradecanoic acid and 906 mg of pre-synthesized $Cd(C_{14}H_{27}O_2)$ were dissolved in 2.35 g of tri-n-octylphosphine oxide at 240 °C. Selenium or tellurium powder, dissolved in tri-n-octylphosphine, was then injected into the reaction mixture at an appropriate time point. The reaction was maintained rigorously at this temperature for 30 min. After cooling to room temperature, 40 mL of methanol and 3 mL of toluene were added. The mixture was subsequently boiled, cooled, and subjected to centrifugation at least three times. The resulting precipitate was then dispersed in pyridine and refluxed under stirring in an oil bath at 90 °C for 12 h. After cooling, the product was washed with n-hexane and isolated by centrifugation.. Finally, the nanocrystals were redispersed in a mixed solvent of pyridine and n-propanol (total volume 3.6 mL), yielding colloidal solutions of CdSe nanocrystals and CdTe nanocrystals, respectively.
